# DAF-16/FOXO Transcription Factor in Aging and Longevity

**DOI:** 10.3389/fphar.2017.00548

**Published:** 2017-08-23

**Authors:** Xiaojuan Sun, Wei-Dong Chen, Yan-Dong Wang

**Affiliations:** ^1^Key Laboratory of Receptor-Mediated Gene Regulation and Drug Discovery, School of Medicine, Henan University Kaifeng, China; ^2^State Key Laboratory of Chemical Resource Engineering, College of Life Science and Technology, Beijing University of Chemical Technology Beijing, China

**Keywords:** DAF-16/FOXO, aging, longevity, lifespan, *C. elegans*

## Abstract

Aging is associated with age-related diseases and an increase susceptibility of cancer. Dissecting the molecular mechanisms that underlie aging and longevity would contribute to implications for preventing and treating the age-dependent diseases or cancers. Multiple signaling pathways such as the insulin/IGF-1 signaling pathway, TOR signaling, AMPK pathway, JNK pathway and germline signaling have been found to be involved in aging and longevity. And DAF-16/FOXO, as a key transcription factor, could integrate different signals from these pathways to modulate aging, and longevity via shuttling from cytoplasm to nucleus. Hence, understanding how DAF-16/FOXO functions will be pivotal to illustrate the processes of aging and longevity. Here, we summarized how DAF-16/FOXO receives signals from these pathways to affect aging and longevity. We also briefly discussed the transcriptional regulation and posttranslational modifications of DAF-16/FOXO, its co-factors as well as its potential downstream targets participating in lifespan according to the published data in *C. elegans* and in mammals, and in most cases, we may focus on the studies in *C. elegans* which has been considered to be a very good animal model for longevity research.

## Introduction

Aging is an inevitable process, commonly defined as gradually functional decline in the time-dependent manner of most living organisms. Characterized by a progressive loss of physiological integrity, it is always accompanied by the risks of many human age-related diseases such as neurodegenerative disorders, cardiovascular diseases, type 2 diabetes and various cancers (Sun et al., 2015). In addition, the world is becoming older as about 20% of the globe will be over 60 years in the near future, which will causes much higher health-care costs as well as more burdens on society (Hansen and Kennedy, 2016). Therefore, how to delay the process of aging and eliminate the potential risk factors for the age-related diseases seem to be urgently required.

As a biological process, aging is not so easily measured because it contains dynamic changes in cells to tissues and organs over time as well as an increased probability of death (Tissenbaum, 2012). Human longevity and healthy aging are complex phenotypes, as they are not only controlled by the heritably genetic factors but also are modulated by environments including living conditions, diet, physical activity, health habits, and psychological factors as well as social interaction. Environments are so rapidly changing that it may cause outer or inner stress conditions for individuals, subsequently requiring the gene regulation work coordinately, which declines during the process of aging, leading to more and more cellular damage. And it has been considered that accumulation of cellular damage is the general cause of aging (Vijg and Campisi, 2008; Gems and Partridge, 2013). Hence, it is not surprising that aging is regarded as the outcome of a balance between damage and repair (Haigis and Yankner, 2010). Lopez-Otin et al. proposed nine relatively comprehensive hallmarks to determine common denominators of aging in different organisms especially for mammalian aging: genomic instability, telomere attrition, epigenetic alterations, loss of proteostasis, deregulated nutrient sensing, mitochondrial dysfunction, cellular senescence, stem cell exhaustion, and altered intercellular communication. Pathological dysfunctions of these nine processes are considered to accelerate aging in mammals while the factors involved in regulation of these hallmarks may contribute to aging (Lopez-Otin et al., 2013; Martins et al., 2016).

The genetic pathways and biochemical processes that modulate aging and longevity are well conserved from budding yeast to the nematode worm *Caenorhabdites elegans* and mammals (Fontana et al., 2010b; Kenyon C. J., 2010). The forkhead transcription factor FOXO as the key downstream regulator that integrates different signals from these pathways plays a crucial role in aging and longevity. Taken incomparable advantages into account, the roundworm *C. elegans* has been considered to be an excellent system for studying molecular mechanisms in regulating animal aging and longevity. Here we discuss the evidence for the role of DAF-16/FOXO in aging and longevity, especially the data in *C. elegans*, which could give clues to the further studies for human aging and longevity.

## General information about DAF-16/FOXO

FOXOs belong to the class O of the Forkhead transcription factors, which is featured by a conserved DNA-binding domain as the Forkhead box or FOX that participates a wide range of important cellular processes such as cell cycle arrest, apoptosis, and metabolism besides its function in stress resistance and longevity (Accili and Arden, 2004).

There are four FOXO genes in mammals: FOXO1 (FKHR), FOXO3 (FKHRL1), FOXO4 (AFX), and FOXO6 sharing high similarity in their structure and function as well as regulation with each other, while invertebrates have only one FOXO gene, named *daf-16* in *C. elegans*, based on the initially isolated dauer defective phenotype when mutated (Albert et al., 1981). *daf-16* is predicted to encode eight distinct transcripts from *daf-16a* to *daf-16h*, of which *daf-16a* and *daf-16d/f/h* are indicated to be the major isoforms involved in dauer arrest and longevity. Human FOXO1 (FKHR), FOXO3 (FKHRL1), FOXO4 proteins share highly similar sequences to DAF-16A and DAF-16D/F/H, especially in Forkhead binding domain. DAF-16A also has the same RxRxxS/T phosphotylation motif with the three FOXOs at their amino-termini, whereas DAF-16D/F/H contains the amino-terminal QxRxxS motif (Kwon et al., 2010; Murphy and Hu, 2013).

As the transcription factor, DAF-16/FOXO contains the DNA binding domain that recognizes a core consensus TTGTTTAC sequence known as the DBE (DAF-16 Binding Element), through which numerous downstream genes called class 1 DAF-16/FOXO targets were discovered (Murphy et al., 2003; Schuster et al., 2010). Besides, DAF-16/FOXO has also been reported to contain DAE (DAf-16 Associated Element) with the GATA site that reverses to CTTATCA sequence over-present in the promoter of the potential target genes, which is responsible for the determination of class 2 DAF-16/FOXO targets as well as some co-transcription factors (McElwee et al., 2004; Tepper et al., 2013).

## DAF-16/FOXO integrates signals of different pathways

### The classic IIS pathway

**Figure 1:**
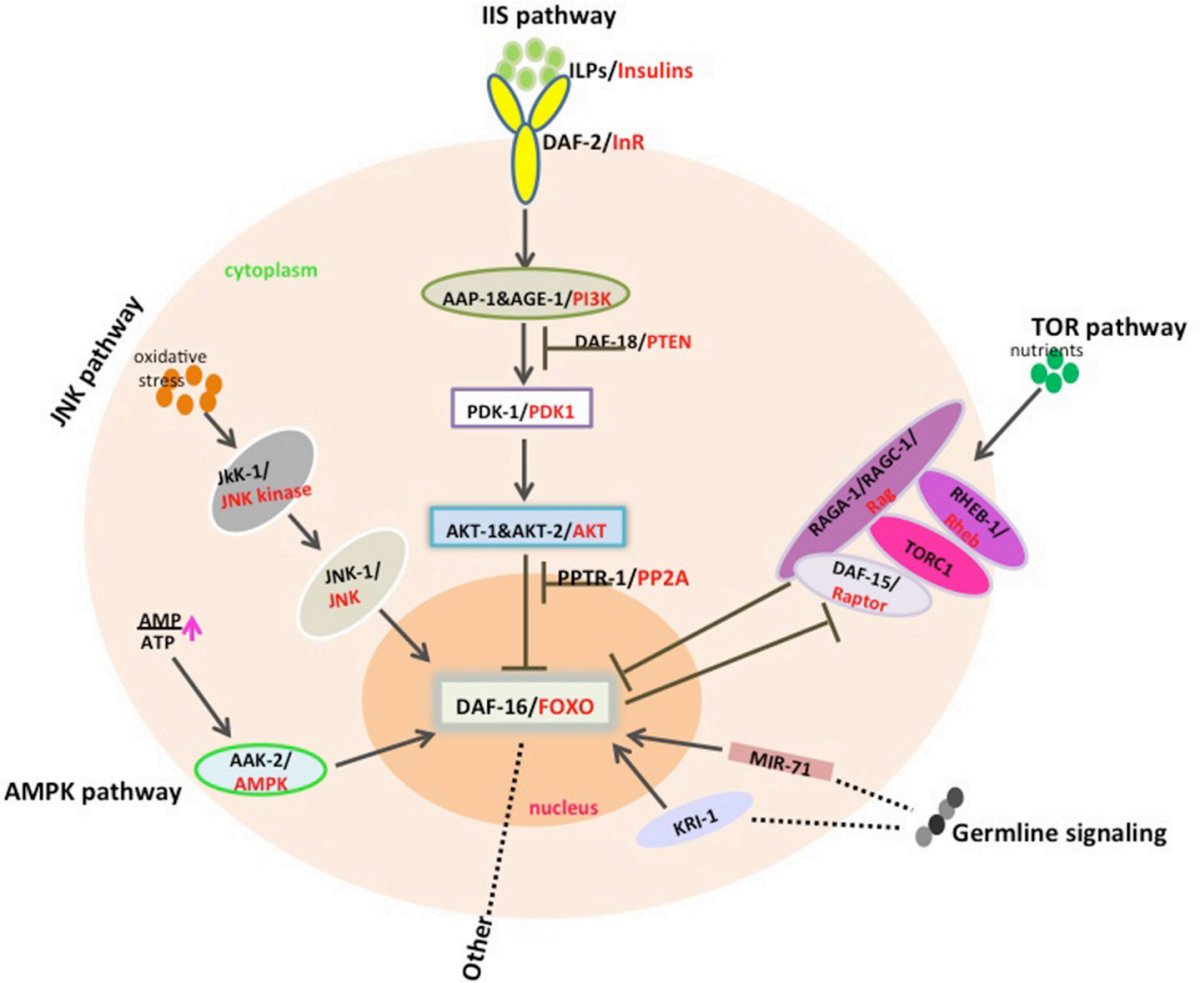
DAF-16/FOXO integrates signals from different pathways to modulate aging and longevity. Insulin-like molecules bind to DAF-2 receptor to lead to the activation of PI3P pathway composed of AGE-1/AAP-1, PDK-1 as well as AKT-1/2, which inhibits DAF-16/FOXO translocation into nucleus by phosphorylation. DAF-18/PTEN and PPTR-1/PP2A negatively regulate the IIS pathway through antagonizing AGE-1/AAP-1 and AKT kinases respectively. Additionally, JNK activity increases under oxidative stress and AMPK is activated upon high AMP/ATP ratios, and both kinases subsequently phosphorylate DAF-16 to promote its activity. Signals from germline, especially in the absence of germline, DAF-16/FOXO would be activated by KRI-1 or by *mir-71* in a cell non-autonomous way. TOR pathway is partially dependent on DAF-16 in the complex of TORC1•DAF-15/Raptor together with Rag GTPases such as RAGA-1/RAGC-1, RHEB-1/Rheb, and in turn, DAF-16/FOXO could also inhibit the expression of the TORC1 coactivator *daf-15/Raptor*.

“Deregulated nutrient sensing” as one of the aging hallmarks to be firstly described to influence longevity, is mainly regulated by the insulin and IGF-1 signaling (IIS) pathway. And this pathway is so highly conserved to modulate aging and longevity across a great evolutionary distance from invertebrates to mammals that the components in every step found in *C. elegans* could be corresponded to the homologs in mice or human (Figure 1) (Fontana et al., 2010b; Kenyon C. J., 2010). The IIS pathway is a signal transduction cascades that consists of insulin-like peptides (ILPs), an insulin/IGF-1 receptor (DAF-2), a phosphoinositide 3-kinase (AGE-1/AAP-1/PI3K), serine/threonine kinases (PDK-1, AKT-1 and AKT-2) and the pivotal downstream Forkhead Box O transcription factor (DAF-16) in *C. elegans*.

DAF-16/FOXO receives phosphorylation from the direct upstream AKT kinases mediated signal transduction response to insulin or IGF and is subsequently sequestered in the cytoplasm by 14-3-3 proteins, which antagonizes FoxO and negatively regulates longevity (Brunet et al., 1999). Therefore, any mutations in the pathway genes including the upstream insulin receptor *daf-2/IGFR*, the signal transducers *age-1, pdk-1* as well as *akt-1*, and *akt-2* all show long-lived phenotypes in the corresponding mutants compared with wild type. And the mechanism is dependent on DAF-16 activity, as *daf-16* null mutations could fully suppress the life span extension phenotypes of the above mutants. Hence, it is not surprising that the negative regulators of the IIS pathway DAF-18/PTEN and PPTR-1/PP2A mutations exhibit short longevity, because DAF-18/PTEN as a lipid phosphatase antagonizes PI3Ks while PPTR-1/PP2A dephosphorylates ATK kinases, which finally affects the distribution of DAF-16/FOXO (Solari et al., 2005; Padmanabhan et al., 2009). Other genes that function upstream of the IIS pathway, such as *unc-31, unc-64, unc-18*, or *unc-13* also modulate lifespan in a *daf-16*-dependent manner (Mukhopadhyay et al., 2006). Any tense conditions that cause inner stress to block the IIS pathway like in the presence of the food restriction or the signals failed to be transduced to DAF-16/FOXO would increase the transcriptional activity of DAF-16/FOXO by inducing the translocation of DAF-16/FOXO to nucleus, which could subsequently promote or repress the expression of downstream targets to trigger the resistance to different kinds of stress and prolong the lifespan of the organisms.

### TOR pathway

Another pathway correlated with nutrition affecting longevity is TOR (target of rapamycin) pathway, which was firstly described in *C. elegans* and was proved evolutionarily conserved later in other organisms (Vellai et al., 2003; Fontana et al., 2010b). Various dietary interventions such as caloric restriction (CR) or dietary restriction (DR) may inactivate TOR pathway to promote lifespan extension. The TOR kinase exists in two distinct complexes, TORC1 containing the coactivator DAF-15/Raptor and TORC2 including RICT-1/Rictor, which function differently in *C. elegans* and in mammalian cells as well (Zoncu et al., 2011). TORC1-mediated longevity via the GTPases RAGA-1/RAGC-1, RHEB-1/Rheb, and DAF-15/Raptor is dependent on DAF-16/FOXO and is also possibly regulated by another transcription factor SKN-1/Nrf in a feedback manner, whereas TORC2/Rict-1 modulates lifespan mainly through SKN-1/Nrf (Lapierre and Hansen, 2012; Robida-Stubbs et al., 2012). It has also been reported that the long-lived phenotype caused by depletion of LET-363/TOR activity through RNAi interference could not be suppressed by daf-16 mutation, suggesting that the function of TOR may be independent of DAF-16, although let-363 RNAi enhanced dauer formation in daf-2(e1370) mutant worms (Vellai et al., 2003). Therefore, it still needs more details to reveal the correlation between TOR-mediated longevity and DAF-16. Additionally, DAF-16/FOXO negatively regulates the expression of the TORC1 coactivator *daf-15/Raptor* (Jia et al., 2004).

TORC1 and IIS have distinct effect on DAF-16 as well as its downstream target genes. IIS inhibits and sequesters DAF-16 in cytoplasm via phosphorylation, so that multiple DAF-16 isoforms accumulate in nuclei once IIS is reduced. In contrast, genetic inhibition of TORC1 increases the *daf-16* mRNA level and leads to only one isoform (DAF-16f) translocate in intestinal nuclei (Robida-Stubbs et al., 2012). In addition, TORC1 may also affect longevity by phosphorylation S6K (S6 kinase), a crucial regulator of mRNA translation that is involved in longevity in *C. elegans* (Kapahi et al., 2010).

### AMP-activated protein kinase (AMPK) pathway

AMPK pathway as an energy-sensing signaling pathway responses to stimuli of decreased energy as well as reduced glucose or leptin levels (Greer et al., 2009). And it is the theoretical basis of dietary restriction regimen that is considered to extend both the mean and maximal lifespan in a wide range of species. AMPK is composed of three subunits including the catalytic α subunit and two β, γ regulatory subunits. In mammalian cells, through binding to AMP, ADP or ATP, the γ subunit could induce a conformational change that allosterically influence the activity of the α subunit which could be activated through phosphorylation by the upstream kinase LKB-1 and CAMKKβ (Solari et al., 2005; Woods et al., 2005). In *C. elegans*, α subunit is encoded by *aak-1* and *aak-2*, while *aakb-1* and *aakb-2* is for β subunit and five isoforms of γ subunits AAKG-1~5. DAF-16 is necessary for AMPK function in oxidative stress resistance and longevity, as the increased longevity caused by overexpression constitutively active (CA) AMPK was reverted when DAF-16 was inhibited (Greer et al., 2007a). Moreover, the mRNA level of *sod-3*, one known DAF-16 target gene involved in both stress resistance and longevity, was highly increased in the CA form of AMPK while it was significantly decreased in the *aak-2* mutant worms, of which the mechanism is probably that AMPK activates the DAF-16 transcription activity by phosphorylation as AMPK could directly phosphorylates DAF-16 *in vitro* via the residues different from the consensus motif phosphorylated by AKT kinases (Greer et al., 2007a). And it is also the same with mammalian FOXO3, indicating it may be conserved throughout evolution (Greer et al., 2007b).

It seems that there is a crosstalk between the IIS pathway and AMPK pathway: previous studies showed that the extension lifespan caused by *daf-2(lf)* could be suppressed by *aak-2* mutation, and one potential explanation is that DAF-16 promotes longevity by stimulating expression of genes encoding AMPK in IIS mutants as DAF-16 could activate expression of *aak-2, aakb-1, aakg-4*, and *aakg-5* according to the previous data, which also suggests the mutual activators between DAF-16, and AMPK (Greer et al., 2007a; Tullet et al., 2014).

### JNK signaling pathway

The JNK (Jun N-terminal kinase) family, a subgroup of MAPK (mitogen-activated protein kinase) superfamily, as a part of signal transduction cascade that is activated by cytokines such as TNF and IL-1, serves as a molecular sensor for various stresses including UV irradiation, ROS (reactive oxygen species), DNA damage, heat, and inflammatory cytokines (Davis, 2000). In *C. elegans*, overexpression JNK or in the *vhp-1* mutant worms that increases JNK activity due to loss of phosphatase activity, showed extension lifespan and resistance to heavy metal toxicity, which may function through phosphorylation DAF-16. Moreover, JNK-1 also promotes the translocation of DAF-16 into nucleus upon heat stress (Mizuno et al., 2004; Oh et al., 2005). Mammalian JNK can directly phosphorylate FOXO4 to enhance its activity (Essers et al., 2004), and JNK may also facilitate FOXO into nucleus by releasing its binding partner 14-3-3 protein via phosphorylation (Sunayama et al., 2005; Yoshida et al., 2005).

JNK pathway has been regarded to act in parallel with the IIS pathway to regulate lifespan before converging onto DAF-16 in *C. elegans* (Oh et al., 2005). Mammalian components of the JNK signaling pathway also interact with the insulin receptor substrate 1 (IRS-1) and the AKT protein kinase. According to the previous studies, JNK inhibited insulin signal transduction through phosphorylation IRS-1 and activated AKT1 via the scaffold protein JIP1 (JNK-interacting protein) that organizes members of the JNK pathway together (Aguirre et al., 2000; Kim et al., 2003).

Collectively, JNK signaling antagonizes IIS pathway to regulate DAF-16/FOXO, although there is a crosstalk between them. JNK directly phosphorylates DAF-16/FOXO to promote its nuclear localization whereas phosphorylated DAF-16/FOXO by IIS pathway AKT inactively retains in the cytoplasm.

### Germline signaling

Reproductive system that may integrate nutrient signaling and communicate with other tissues through germline to affect aging has been observed in *C. elegans*, flies as well as in mice, indicating a conserved regulation mechanism across different organisms (Kenyon C., 2010). And it has been reported that the lifespan could be extended by 40–60% if the germline precursor cells were removed or the germline stem cell division were prevented in *C. elegans* (Hsin and Kenyon, 1999; Arantes-Oliveira et al., 2002). A steroid hormone pathway that includes the key components DAF-36/NVD, DAF-9/CYP27 as well as DAF-12/NHR is required for lifespan extension in response to germline loss, and DAF-12/NHR and DAF-9/CYP27 probably form complex with DAF-16/FOXO to function, although the detailed mechanisms remain to be further determined (Dowell et al., 2003; Hansen et al., 2013). In ablated-germline worms, transcription factor DAF-16/FOXO was activated and primarily translocated to the intestinal nucleus, which was found to be regulated by an intestinal ankyrin repeat protein KRI-1 or by microRNAs such as *mir-71* in a cell non-autonomous way (Berman and Kenyon, 2006; Boulias and Horvitz, 2012). Once DAF-16/FOXO was translocated into nucleus, it would activates some downstream targets that may be involved in fat metabolism such as the lipases *lipl-4* and *lips-17*, the direct regulator for longevity in germline-less animals (Wang et al., 2008; McCormick et al., 2012). In addition, DAF-16/FOXO also modulates numerous genes that take part in steroid hormone metabolism such as the steroid hormone dehydrogenases cytochrome P450s (Hansen et al., 2013), suggesting an indirect regulation manner.

Several components of the IIS pathway including the negative regulator DAF-18/PTEN (Berman and Kenyon, 2006), the cofactor SMK-1/SMEK-1 (Wolff et al., 2006) and the transcription factor HSF-1 (Hansen et al., 2005) are also responsible for lifespan extension upon germline loss besides that germline ablation further extends lifespan of long-lived *daf-2* mutants, indicating a connection between IIS pathway, and germline signaling. However, the distribution of DAF-16/FOXO in tissues shows different under the regulation of the two pathways. The IIS pathway mainly affects DAF-16/FOXO in both neuronal and intestinal cells, whereas germline ablation leads to DAF-16/FOXO translocation to the nucleus primarily in intestinal cells (Lapierre and Hansen, 2012).

There also exist other components that function dependent on DAF-16/FOXO. Example, in *C. elegans*, there is a special developmental larval stage called dauer, and the worms arrest at the dauer diapause to live longer upon tense conditions, which is mainly regulated by IIS pathway as well as TGF-β like signaling pathway composed of TGF-β-like ligand DAF-7, the Type 1 and 2 receptors DAF-1 and DAF-4, and the downstream DAF-3 Smad and DAF-5 Sno/Ski (Fielenbach and Antebi, 2008; Gumienny and Savage-Dunn, 2013). According to the genetics epistasis analysis, these two pathways may function in parallel. The TGF-β like signaling pathway could also regulate DAF-16 localization and the DAF-16 target gene *sod-3* transcription (Shaw et al., 2007). In addition, PDP-1, genetically function at the level of the R-SMAD proteins DAF-16, and DAF-8 in the TGF-β like signaling pathway, also promotes DAF-16 nucleus localization and transcriptional activity, therefore, it was considered to link TGF-β, and IIS pathways to modulate longevity and development (Narasimhan et al., 2011).

### Regulations of DAF-16/FOXO

Temporal regulation of DAF-16/FOXO expression is conserved. *FOXO3* and *FOXO4* transcript are undetectable at very young stage but increased in the duodenum in elder rats (Huang et al., 2011), and the human *FOXO1* mRNA level shows significantly enriched in old individual muscles as well (Buford et al., 2011), while *C. elegans daf-16d/f* transcription expression also exhibits dramatically increased during the young adult stage, and this upregulation of *daf-16d/f* expression is responsible for longevity. So far, *elt-2* (GATA transcription factor) and *swsn-1* (core subunit of SWI/SNF complex) have been identified to modulate *daf-16d/f* mRNA level besides that TORC1 negatively regulate the *daf-16* gene transcription expression (Bansal et al., 2014). However, the crucial roles of DAF-16/FOXO in aging and longevity as well as other cellular processes are mainly through post-translational modifications including phosphorylation, acetylation, methylation and ubiquitination.

DAF-16/FOXO is prone to be phosphorylated by a group of protein kinases at different sites in response to external or internal stimuli, which leads to the alteration of the subcellular localization, protein stability, DNA-binding properties and transcriptional activity. As the primary substrate of AKT/PKB, the phosphoacceptor sites of *C. elegans* DAF-16 are conserved in mammals, and phosphorylated DAF-16/FOXO by ATK/PKB shows inhibitory transcription activity with retention in cytoplasm (Paradis and Ruvkun, 1998; Brunet et al., 1999; Kwon et al., 2010). In contrast, another *C. elegans* AGC family serine-threonine kinase SGK-1 exhibits opposite effect to influence longevity as well as stress resistance in a DAF-16 -dependent manner without affecting DAF-16 subcellular localization (Chen et al., 2013), which is different in mammalian cells (Brunet et al., 2001). DAF-16/FOXO also undergoes inhibitory phosphorylation by protein kinases such as AMPK, ERK, CDK2, GSK3, CK1, and IKK when exposed to stimuli (Hu et al., 2004; Huang et al., 2006; Greer et al., 2007a; Yang et al., 2008; Huo et al., 2014), whereas DAF-16/FOXO is usually activated and translocated in nucleus via phosphorylation in the presence of CDK1, JNK, MST1 as well as CAMKII (Essers et al., 2004; Oh et al., 2005; Lehtinen et al., 2006; Yuan et al., 2008; Tao et al., 2013).

DAF-16/FOXO also undergoes acetylation to mediate numerous biological processes besides aging and longevity under the control of histone acetyltransferase CBP/P300 (van der Heide and Smidt, 2005). And acetylated DAF-16/FOXO is more likely to localize to cytoplasm with the abolished DNA-binding capacity (Matsuzaki et al., 2005). On the other hand, Histone deacetylases (HDACs) SIR2/SIRT1 has been demonstrated to be required for lifespan extension possibly through its deacetylase activity and modulation the downstream targets of DAF-16/FOXO (Bordone and Guarente, 2005; Guarente and Picard, 2005; Wang and Tissenbaum, 2006).

The protein stability of DAF-16/FOXO is determined by the ubiquitin-proteasome pathway. Several ubiquitin E3 ligases such as MDM2, SKP2, COP1, and CHIP are responsible for the ubiquitination and degradation of FOXOs (Huang et al., 2005; Kato et al., 2008; Yang et al., 2008; Li et al., 2009). Additionally, Phosphorylation by ERK or IKK also contributes to the FOXOs degradation (Hu et al., 2004; Yang et al., 2008).

DAF-16/FOXO could also be methylated by the protein arginine methyltransferase PRMT1. By blocking the phosphorylation of DAF-16 via AKT, *C. elegans* PRMT1 plays an important role in longevity and stress tolerance through direct methylation DAF-16 (Takahashi et al., 2011), which is similar to what has been discovered in mammalian cells (Yamagata et al., 2008).

### Targets of DAF-16/FOXO

For the crucial role in aging and longevity, it is essential to determine the targets of DAF-16/FOXO transcription factor that might presumably function by activating or inhibiting its downstream genes. By using various high throughout techniques including microarray (McElwee et al., 2003; Murphy et al., 2003), proteomics (Dong et al., 2007), and DamID (DNA adenine methyltransferase identification) (Schuster et al., 2010), numerous direct or indirect targets were identified, and about 109 DAF-16 direct targets in *C. elegans* were indicated (Li and Zhang, 2016). Since DAF-16 is involved in multiple biological processes besides the regulation in aging and longevity, so do these downstream targets. Here, we just summarized the potential targets in the list that have been reported to influence aging and longevity (see in Table 1) (Oh et al., 2006; Tullet, 2015; Li and Zhang, 2016), and the homologs in human are illustrated based on the descriptions in Wormbase.

**Table 1:** The DAF-16 direct targets that are involved in longevity.

***C. elegans* gene**	**Description**	**Longevity influence**	**Homologues in human**
*aco-2*	ACOnitase	Anti-longevity	ACO2
*C01B7.1*	Zinc-finger domain (C2H2 type)	Pro-longevity	
*daf-7*	Abnormal DAuer Formation	Anti-longevity	
*din-1*	DAF-12 Interacting Protein	Pro-longevity	spen
*dod-17*	Downstream Of DAF-16(epoxide hydrolase)	Anti-longevity	EPHX1
*egl-10*	G protein signaling component	Anti-longevity	RGS6/7
*F42G10.1*	Neprilysin-B-like gene (peptidase family M13)	Pro-longevity	
*gpd-2*	Glyceraldehyde 3-Phosphate Dehydrogenase	Anti-longevity	Glyceraldehyde-3-phosphate dehydrogenase
*icl-1*	Isocitrate lyase/malate synthase		
*lab-1*	Lim domain binding protein	Pro-longevity	
*lars-2*	Leucyl Amino-acyl tRNA Synthetase	Anti-longevity	LARS2
*lgg-1*		Pro-longevity	LC3
*lin-2*	MAGUK family protein kinase	Anti-longevity	GASK
*mdh-1*	Malate DeHydrogenase	Pro-longevity	Mdh2
*mrpl-12*	Mitochondrial Ribosomal Protein	Anti-longevity	mRpL12
*nnt-1*	Nicotinamide Nucleotide Transhydrogenase	Pro-longevity	
*pck-2*	Phosphoenolypyruvate CarboxyKinase	Anti-longevity	PCK1/2
*prdx-3*	PeRoxireDoXin	Pro-longevity	prdx3
*sams-1*	S-adenosyl methionine synthetase	Anti-longevity	MAT1A/MAT2A
*sca-1*	Sarco-ER calcium ATPase	Anti-longevity	ATP2A1
*ubh-4*	UBiquitin C-terminal Hydrolase	Anti-longevity	UCHL5
*zfp-1*	Zinc-finger protein	Pro-longevity	MLLT6/10

## Conclusions and perspectives

Future exploration needs unravel the precise roles of DAF-16/FOXO in aging and longevity so that it would provide more implications to delay or prevent aging and age-dependent diseases.

## Author contributions

XS wrote the manuscript. YW and WC reviewed and revised the manuscript.

### Conflict of interest statement

The authors declare that the research was conducted in the absence of any commercial or financial relationships that could be construed as a potential conflict of interest.
